# Oxygennesis for the Instantaneous Production of Pure Oxygen without Heat

**Published:** 1864-06

**Authors:** J. Robbins


### OXYGENNESIS FOR THE INSTANTANEOUS PRODUCTION OF PURE OXYGEN WITHOUT HEAT.

By MR. J. ROBBINS.

From the time of the discovery of oxygen to the present, perhaps no subject has so much engaged the attention of chemists as the production of it at a cost sufficiently low to be employed in the arts, for the reduction of metals and other operations requiring a high temperature, and also for the purposes of illumination, the light obtained by it vieing in splendor with the sun's rays. This desirable object has yet to be accomplished, and may be regarded as a prize to be won by some future happy discoverer. It seems surprising, since Nature has provided us so bountifully with this substance, and presented it in such a variety of combinations, that, up to the present time, there should be no known method of separating, except at such a cost which excludes it for the purposes just enumerated.

In Gmelin's "Chemistry" six processes are given for the production of oxygen:—

1st. By heating chlorate of potash to low redness. As this process is slow and tedious, a small quantity of oxide manganese is usually mixed with the salt, which greatly facilitates the decomposition, and the evolution of gas is both rapid and abundant. According to the authority just named, manganese is often mixed with carbonaceous matter, which passes over as carbonic acid, but another impurity I may mention is the presence of chlorine, which, I believe, may always be detected when oxygen is obtained by this method.

2d. By ignition of red oxide of mercury. The presence of hyponitric acid may be feared in oxygen so obtained.

3d. By strong ignition of oxide of manganese.

4th. By heating manganese with an equal weight of oil of vitriol.

5th. By ignition of nitrate of potash. This salt, when heated above its melting point, is converted by the loss of two equivalents of oxygen into nitrite of potash; on a further increase of temperature both nitrogen and oxygen pass off, consequently the product is always contaminated with nitrogen, which increases as the action proceeds.

6th. By the action of sulphuric acid on bichromate of potash. Three parts bichromate potash and four parts sulphuric acid are heated together in a capacious retort. An evolution of oxygen gas easy to regulate is the result. In this experiment we might congratulate ourselves if the process is conducted to the end without a fracture of the retort.

Of the six processes just described, two only are now used, viz.: chlorate of potash with or without manganese, and manganese alone. More recently other processes have been recommended, one of which, by heating together nitrate of soda and oxide of zinc, has been patented. From this mixture oxygen is said to be produced at a cheaper rate than by any other method at present known. Unfortunately for the value of this discovery, the product is contaminated with a considerable percentage of nitrogen. M. Kuhlman, of Lille, discovered and published an ingenious and beautiful process for the production of oxygen by means of baryta. He found that by passing a current of common air through caustic baryta heated to dull redness, peroxide of barium was formed, which, on an increase of temperature, is resolved into oxygen gas and caustic baryta; the latter ready again to perform its part in a similar operation. The idea naturally suggested itself that the means were now at hand for getting oxygen from the atmosphere in any quantity at a small cost. This method, although so promising, has been for the present abandoned; it was found that, after a few operations, either from a morecular change, or from the silica or other impurities, a sort of glass or fusion resulted on the surface, the baryta then refusing to act again.

We now come to the consideration of the new method for the generation of oxygen recently introduced by myself. The process or the compound employed in it has been named *oxygennesis.* It will have doubtless been observed by you that, in all the processes hitherto known, a high temperature is necessary, and until that point is reached, no product whatever is obtained; this fact we may consider as the chief difficulty experienced in the preparation of oxygen, and more especially so when sulphuric acid is used. If, for example, by the mere addition of sulphuric acid to bichromate of potash in the cold, we could get the same results which are obtained by the application of heat, this process, instead of being thrust in the rear, would have taken front rank.

Oxygennesis, therefore, stands alone as a novel and the only mode we possess for producing oxygen without the application of heat. The mode of using this compound is extremely simple. We have only to take some of this powder, place it in a glass flask or bottle provided with an exit tube, pour on any of the dilute mineral acids, and we have immediately oxygen evolved in a similar way, and with as much facility as hydrogen is obtained from zinc or carbonic acid from a carbonate.

The composition of this compound is extremely simple, merely peroxide of barium, and bichromate potash. Not so the chemical changes resulting from an addition of an acid. Peroxide of barium, on addition of sulphuric acid, is resolved into sulphate of baryta and peroxide of hydrogen, and it is from this sometimes so-called oxygenated water we get this curious and interesting chemical reaction. Whenever peroxide of hydrogen and chromic acid are brought into contact with each other, instantaneous decomposition is the result, the chromic acid is reduced to sequioxide of chromium, and the peroxide of hydrogen to water; at the same time pure oxygen derived from both, those substances is disengaged. The theory of this very interesting reaction is not, I believe, well understood, and I know only one way of explaining it, that is, on the ozone and antozone theory of Brodie. According to that theory, oxygen exists in three different states or conditions, viz.: ozone, antozone, and ordinary oxygen, and whenever ozone and antozone (which may both be considered more or less active) are brought together, they unite and neutralize each other, as it were forming passive or common oxygen.

To return to the composition of the powder, we are not compelled to use precisely those ingredients mentioned, but may substitute analogous compounds. Peroxide of barium might be replaced by any other peroxide capable of forming binoxide of hydrogen, of which there are several—peroxides potassium, sodium, strontium, and calcium—but all these at the present time are practically useless, peroxide of barium being the only one that can be easily and cheaply prepared. Bichromate of potash may be substituted by manganate or permanganate of potash, binoxide of manganese or binoxide of lead; the cost of the two first-mentioned forbids their present use, and the one selected is by far preferable to the others. With regard to the acids, either of the mineral class will do, but I prefer a mixture of dilute sulphuric and hydrochloric acids.

The next question demanding our notice is, in a commercial point of view, a most important one; however much this method may be admired for its simplicity, and the ease with which the operation may be conducted, its ultimate success or failure must depend on its cost. Can the oxygennesis, therefore, be manufactured and sold at a price sufficiently low to make it an article of commerce? I believe it can be, and be made available for all purposes wherever oxygen is required to the extent of some gallons. One of the ingredients of this compound, peroxide of barium, has never yet been produced and sold as a commercial article, and from the trouble of making a small quantity, but few even practical chemists care to prepare it for themselves. It can hardly, therefore, be expected that a compound of this nature can at once be manufactured and sold at a price it must ultimately be reduced to, if extensively used and produced in quantity. 5s. per pound, the price hitherto charged, would, I admit, be a barrier to its general adoption; but I am happy to say we have now made the necessary arrangements to lessen the cost of production, and have at the same time reduced the price.

Some of the baryta compounds are found abundantly in nature, and are of but small value in the market, but up to the present time but few uses have been made of them; they now promise a much more extensive application. Mr. Kuhlman has, perhaps, done more than any one else to develop their uses and value in the arts, and in the *Chemical News*, November 28, 1863, will be found some interesting extracts relating to them from Dr. Hofmann's report on chemical products and processes of the International Exhibition.

I shall trespass a little farther on your time to make a few remarks on one of the various applications of oxygen, which may be of some interest to the medical profession and to pharmaceutical chemists. I mean the employment of that body as a therapeutic agent by inhalation. For that purpose this ready method for producing the gas promises to be of great value. Towards the end of the last and the beginning of the present century, vital air, as oxygen was often and not inappropriately called, was used largely in this country and on the continent. In this country we find the names of Drs. Beddoes, Hill, Thornton, and other physicians. Dr. Hill used it for more than 25 years, and Dr. Thornton was quite eminent for his successful application of it. At the present time, the desire by medical men for the administration of oxygen has revived both here and abroad. Two papers have recently been read, to be followed by others on the same subject, before the Academy of Sciences at Paris, by Messrs Demarquay and Leconte. The experiments and observations of those gentlemen appear to have been very numerous and carefully made both on animals and on man, in disease and in health, and the conclusion they arrive at is, that oxygen is a valuable curative agent. If so good, then, as a remedy when its value was once known, what caused it to become and continue so long neglected? The explanation is, I think, not difficult. In the first place, when this body was discovered, too much was expected from it. The first *furore* for its employment arose from the simple experiment which showed its power of rekindling an expiring match, and as oxygen is the essential element of our existence, it was supposed it might, in a similar way, rekindle the expiring vital spark. The more imaginative were elated at what they considered a discovery, so long dreamt of and so earnestly sought after by the alchemist. But oxygen is not the elixir vitæ. It will not restore grey hair to its original color, nor make an old man young. The difficulties and expense attending its administration may also be considered other reasons for its non-employment. Of what use was it for a medical man to order that which the patient could not get supplied? A physician, therefore, having faith in the remedy, was compelled to lay himself out especially for it, become an oxygen doctor, and prepare and administer the remedy himself. These difficulties now no longer exist.

We have on the table an oxgyen inhaler and generator, made according to the suggestions of Dr. Richardson. The generation of the gas is by this method so easy and so simple that patients can prepare their own dose, or, if need be, the nurse after one lesson can as well undertake the operation as any other duty she may be required to perform.

Physicians who may wish to employ this remedy may now prescribe it with no more hesitation than they would prescribe a black draught or a calomel pill.

Dr. Squire wished to enquire whether Mr. Robbins' invention had not been patented; if so, he doubted the value of the patent, for Prof. Brodie had pointed out in 1851, that when peroxide of barium was treated with an acid solution of bichromate of potash oxygen was evolved with great regularity. Faraday had mentioned the same thing in one of his lectures on ozone at the Royal Institution. Dr. Squire also objected to the name oxygennesis, and thought it ought rather to be called oxyexodus, as the oxygen was eliminated, not created.

Mr. C. H. Wood said that the reactions of peroxide of barium and bichromate or permanganate of potash were well known as scientific facts, but did not consider that that effected the value of Mr. Robbins' patent. The great objection to Mr. Robbins' process was the cost of the oxygen, and he was glad to hear that this was likely to be reduced. He did not consider that the process was easier than that for obtaining oxygen from chlorate of potash and manganese; indeed, he thought the latter the easier operation. He wished to know how Mr. Robbins decided that oxygen from chlorate of potash and manganese was contaminated with chlorine and not by ozone. The late Mr. Witt had shown that oxygen obtained in this way always contained a variable proportion of ozone. He (Mr. Wood) did not consider ozone objectionable for inhalation, for he thought that whatever good was effected by oxygen, when inhaled, must be due to the ozone which gave active properties to oxygen.

Prof. Redwood said that Mr. Crace Calvert had pointed out that oxygen from chlorate of potash and peroxide of manganese always contained chlorine or an oxide of it, and he did not think that the gas from this source was fit to administer. He freely gave his testimony to the value of the process Mr. Robbins had introduced. The process was certainly easy, though it was not calculated for chemists, but would answer well when oxygen was wanted for medical purposes.

Mr. Robbins, in reply, said that he was not aware of Mr. Brodie's experiments, and had not heard of them, although he had spoken to many chemists on the subject. He considered it a great boon to be able to get oxygen by a cold process, and at all events, still considered the application new. Authorities differed as to the value of oxygen as a curative agent, but the most recent experiments of Demarquay and Leconte seemed to prove that it possessed great remedial power. Under these circumstances, it was well to have a means of procuring oxygen as easily as making a cup of tea. With regard to Mr. Wood's doubt about the presence of ozone instead of chlorine, he might say that, in conjunction with Mr. Brown, now of the War Department, he had examined many specimens of oxygen obtained from the chlorate and manganese for ozone, but in all cases found chlorine instead. In his (Mr. Robbins') process the oxygen was well washed.

Mr. Wood said that washing would not remove ozone.

Mr. Robbins replied that Faraday had showed that ozone could be removed by washing, and he himself had found ozone made by means of phosphorus in the ordinary way disappear after shaking with water well for an hour.

The Chairman said he considered Mr. Robbins' an elegant way of procuring oxygen for medical purposes, which had the recommendation of cheapness when the oxygen was considered as medicine.—*Chemical News, London, March* 12, 1864, from *Trans. Lond. Pharm. Society.*

